# Phylogeny, sequence-typing and virulence profile of uropathogenic *Escherichia coli* (UPEC) strains from Pakistan

**DOI:** 10.1186/s12879-019-4258-y

**Published:** 2019-07-12

**Authors:** Ihsan Ali, Zara Rafaque, Ibrar Ahmed, Faiza Tariq, Sarah E. Graham, Elizabeth Salzman, Betsy Foxman, Javid Iqbal Dasti

**Affiliations:** 1 Department of Microbiology, Faculty of Biological Sciences, Quaid-i-Azam University, Islamabad, 45320 Pakistan; 2 Alpha Genomics (Pvt) Ltd, Islamabad, Pakistan; 3 Department of Biophysics, University of Michigan, Ann Arbor, MI 48109 USA; 4 Department of Epidemiology, School of Public Health, University of Michigan, Ann Arbor, MI 48109 USA; 5 Department of Medical Laboratory Technology (MLT), the University of Haripur, Abbottabad, Pakistan

**Keywords:** ST131, VF genes, ESBL, UPEC, MDR

## Abstract

**Background:**

*Escherichia coli* lineage ST131 predominates across various spectra of extra-intestinal infections, including urinary tract infection (UTI). The distinctive resistance profile, diverse armamentarium of virulence factors and rapid global dissemination of ST131 *E. coli* makes it an intriguing pathogen. However, not much is known about the prevalence and genetic attributes of ST131 lineage in Pakistan.

**Methods:**

We estimated prevalence and genetic attributes of *E. coli* ST131 isolates causing UTI among 155 randomly selected samples. Samples were analyzed for phylogenetic grouping, O-typing and *fum*C/*fim*H typing. Isolates were further tested for the ESBL and virulence factors using PCR.

**Results:**

Overall, 59% of the UPEC isolates belonged to the phylogenetic group B2, followed by D = 28%, B1 = 8% and A = 5%. Among 18 different Sequence-types, ST131 was the dominant lineage (*n* = 71; 46%) out of which 72% of the isolates were assigned to the phylogenetic group B2, while 61% adhered to the serogroup O25b. *Fum*C/*fim*H typing confirmed 49% of the ST131 as *H30* sub-types. In this study, significant numbers of the identified ST131 isolates were MDR and 42% showed ESBL phenotypes, out of which 37% carried *bla*-_CTX-M-15_. Moreover, different virulence factors were detected in following percentages: *fim*H,155(100%), *iut*A 86 (55%), *feo*B 76 (49%), *pap*C 75 (48%), *papGII *70 (45%), *kpsMTII* 40 (26%), *pap*EF 37 (24%), *fyu*A 37 (24%), *usp* 22 (14%), *pap*A 20 (13%), *sfa/foc*20 (13%), *hly*A 18 (12%), *afa* 15 (10%), *cdt*B 11 (7%), *papGI* 6 (4%), *papGIII* 6 (4%), *kpsMTIII* 4 (3%) and *bma*E2 (1%).

**Conclusion:**

Conclusively, this study provides important insight into the genetic and virulence attributes of pandemic MDR ST131 strains involved in UTIs. It also highlights higher prevalence of ST131-O25b-H30 UPEC isolates in patients, which was previously unreported from this part of globe.

## Background

Extra-intestinal *E. coli* is the major cause of urinary tract infections and resistance among UTI strains has been mounting against different antibiotics, including trimethoprim-sulfamethoxazole, fluoroquinolones extended spectrum cephalosporins and amoxicillin clavulanic acid [1–3]. Due to the emergence of specific clonal groups such as ST131, global dissemination of fluoroquinolone-resistance was highlighted across different geographical regions [4–6]. Clonal group ST131 predominates across various spectra of infections including cystitis, pyelonephritis, bacteremia, meningitis, septic shock, epididymo-orchitis and osteoarticular infection. [7, 8]. In addition, ST131 strains harbor diverse armamentarium of virulence factors and their genetic homogeneity regarding virulence potential and resistance profile has been widely endorsed [8]. Notably, a subgroup of ST131 strains, known as *H30-*Rx has remarkable tendency to encode *bla*-_*CTX-M-15*_ gene [7, 9, 10]. In the current scenario of global urgency related to the antibiotic resistance, underlying epidemiological factors related to the fitness and fast emergence of ST131 across different regions are under intensive scrutiny. However, in Pakistan phylogenetic grouping, sequence types, virulence attributes and antibiotic susceptibility profile of UPEC strains remains unexplored [11, 12]. Therefore, data related to the clonal types and resistance profile of the strains involved in urinary tract infections in Pakistan is extremely scarce. This study fills the gap and provides important insights into the genetic and virulence attributes of pandemic MDR ST131 strains involved in UTIs in Pakistan.

## Methods

### Sample collection and antibiotic susceptibility testing

Altogether *n* = 155 identified uropathogenic *E. coli* (UPEC) were collected during the period of August 2012 to August 2014, from Pakistan Institute of Medical Sciences. Ethical Review Board (ERB) of Pakistan Institute of Medical Sciences approved this study. Ethical Review Board approved verbal consent taken from all the patients. Important patient data such as name, age, gender, location was recorded and unique identification number were assigned to each patient. Samples were from community-acquired urinary tract infections. Antibiotic testing and phenotypic detections of ESBL were performed by disc diffusion methods according to the guidelines CLSI, 2014 [13]. Isolates were tested for the susceptibility to 12 different classes of antibiotics including β-lactamase inhibitors (piperacillin tazobactam, amoxicillin-clavulanic acid), cephalosporins (ceftazidime, cefotaxime, ceftriaxone), fluoroquinolone (ciprofloxacin, levofloxacin), aminoglycosides (amikacin), trimethoprim sulfonamides, nitrofurantoin, and fosfomycin (BIOANALYSE, Turkey). Control strain *E. coli* ATCC 25922 was used in this assay.

### Phylogeny, serotyping, and *fum*C/*fim*H typing

We used the procedure reported by Clermont et al, 2000 to perform phylogenetic analysis of 155 isolates [14]. *Fum*C/*fim*Htyping (CH typing) was performed as previously described [15]. Briefly, PCR amplifications were carried out in 25 μl (12.5 μl GoTaq DNA polymerase (Promega), 7.5 μl water, 1 μl bacterial DNA, 2 μl of each forward and reverse primers). The amplified products were analyzed on 2% agarose gel. The confirmed PCR products were purified using PCR purification kit (QIAquick, QIAGEN) and all the amplified DNA fragments were sequenced (ABI 3130, Perkin-Elmer Applied Biosystems, Foster City, California). The forward and reverse sequences were aligned, trimmed off using Codon Code Aligner and results were compiled according to the standard procedures [15, 16]. Additionally, by targeting 347 bp of *pab*B gene fragment, clonal group ST131 was scrutinized for serogroup O25b [17]. Previously typed O25b-ST131 strains and K-12 *E. coli* were included as experimental controls in this study.

### Detection of β-lactamases and virulence factor genes

In order to detect extra-chromosomally encoded ESBL factors, plasmid DNA was isolated by commercially available kit (Thermo-Scientific Gene Jet plasmid Miniprep Kit). ESBL factors including *bla*_**-**TEM_, *bla*_-SHV_ and genes *bla*_-OXA_*, bla*_-PSE_ were PCR amplified as described elsewhere [18]. Amplified products were then purified (Gel Band Purification Kit, Amersham, USA) and sequencing was done by automated DNA sequencer (ABI 3130, Perkin-Elmer Applied Biosystems, Foster City, California). Sequences were reported to the Gene Bank database (accession number; KX171170–171195). PCR amplifications and sequencing of *bla*_*-*CTX-M_ allele was carried out*, bla*_-CTX-M_ types were determined by comparing DNA sequences available in the database [19]. A total of 18 different virulence factors **(**VF) corresponding to the main classes of extra-intestinal virulence associated genes (VAGs) including adhesins, toxins, siderophores, capsular proteins and uropathogenic-specific protein (*usp*) were scrutinized in all 155 isolates. VF genes were amplified by previously reported sets of primers and amplification conditions [20].

### Statistical analysis

The statistical analysis was performed using Graph Pad Prism, version 7. Both Chi square and Fisher exact tests were used to assess differences by assuming cut-off value of *P <* 0.05 as significant.

## Results

### Phylogeny and sequence typing

Overall, phylogenetic group B2 showed highest representation, 92(59%) followed by D 43 (28%), B1 12 (8%) and A 8(5%) (Table 1). Eighteen different sequence types (STs) of 152 isolates were confirmed, constituting 98% of all the isolates; the remaining 2% of the isolates were un-typeable. Clonal group ST131 comparised 71(46%) of all the isolates, followed by two other lineages, ST405 28(18%) and ST168 16(10%) (Table 2). Majority of the ST131 strains 51(72%) belonged to the phylogenetic group B2, while 43(61%) were assigned to serogroup O25b. CH typing confirmed 35(49%) as ST131-*H*30 sub-group of strains, out of which 22(31%) belonged to the serogroup O25b.

**Table 1:** Distribution of ESBL factors, antibiotic resistance and VF genes in MDR UPEC

Numbers and percentagesof isolates and their respective traits (*n* = 155)						
Resistance traits	Total Isolates (*n* = 155)n (%)	Group A (*n* = 8)n(%)	Group B1 (*n* = 12) n(%)	Group B2 (*n* = 92) n(%)	Group D (*n* = 43)n(%)	*p* value
ESBL phenotypes	65(42)	1(13)	3(25)	36(39)	25(58)	0.0268
*bla*CTX-M-15	57(37)	1(13)	3(25)	32(35)	21(49)	0.1333
*bla*TEM	23(15)	1(13)	3(25)	13(14)	6(14)	0.7823
*bla*SHV	6(4)	00	00	3(3)	3(7)	0.0907
*bla*OXA	10(6)	00	1(8)	4(4)	5(12)	0.3610
*bla*PSE	1(0.6)	00	00	1(1)	00	0.8783
Piperacillin tazobactam	7(5)	00	1(8)	4(4)	2(5)	0.8514
Ceftazidime	96(62)	3(38)	7(58)	54(59)	32(74)	0.1483
Cefotaxime	101(65)	3(38)	7(58)	59(64)	32(74)	0.2028
Ceftriaxone	99(64)	3(38)	8(67)	59(64)	29(67)	0.4001
Ciprofloxacin	95(61)	6(75)	7(58)	56(90)	26(60)	0.4001
Levofloxacin	97(63)	7(88)	8(67)	56(90)	26(60)	0.4929
Amikacin	7(5)	1(13)	00	5(5)	1(2)	0.4920
Gentamicin	47(30)	4(50)	6(50)	24(26)	13(30)	0.2171
Amoxicillin-clavulanic acid	111(72)	5(63)	7(58)	66(72)	26(60)	0.5224
Trimethoprim sulfonamides	130(84)	7(88)	11(92)	77(84)	35(81)	0.8461
Nitrofurantoin	9(6)	1(13)	1(8)	6(7)	1(2)	0.6075
Fosfomycin	15(10)	1(13)	2(17)	8(9)	4(9)	0.8370
*fimH*	155 (100)	8 (100)	12 (100)	92 (100)	43 (100)	> 0.9999
*papA*	20 (13)	1 (13)	1 (8)	12 (13)	6 (14)	0.9659
*papC*	75 (48)	2 (25)	8 (67)	43 (47)	22 (51)	0.3092
*papEF*	37 (24)	0	6 (50)	19 (21)	12 (28)	0.0476
*papGI*	6 (4)	0	0	3 (3)	3 (7)	0.5699
*papGII*	70 (45)	2 (25)	7 (58)	37 (40)	24 (53)	0.1695
*papGIII*	6 (4)	0	0	4 (4)	2 (5)	0.8177
*sfa/foc*	20 (13)	2 (25)	1 (8)	13 (14)	4 (9)	0.5968
*Afa*	15 (10)	0	1 (8)	10 (11)	4 (9)	0.7919
*bmaE*	2 (1)	0	1 (8)	1 (1)	0	0.1466
*fyuA*	37 (24)	1 (13)	2 (17)	21 (23)	13 (30)	0.5882
*iutA*	86 (55)	5 (63)	5 (42)	52 (57)	24 (56)	0.7701
*feoB*	76 (49)	3 (38)	6 (50)	40 (43)	27 (63)	0.1852
*kpsmtII*	40 (26)	0	4 (33)	21 (23)	15 (35)	0.1438
*kpsmtIII*	4 (3)	0	1 (8)	3 (3)	0	0.3765
*Usp*	22 (14)	2 (25)	2 (17)	14 (15)	4 (9)	0.6256
*hlyA*	18 (12)	1 (13)	3 (25)	6 (7)	8 (19)	0.0908
*cdtB*	11 (7)	0	3 (25)	6 (7)	2 (5)	0.0758

Distribution of resistance and virulence traits among different phylogroups of uropathogenic *E. coli* (*N* = 155). The *p* values were calculated by comparing different traits among phylogroups

**Table 2:** Distribution of MDR and fluoroquinolone resistant MDR strains in different phylogroups and ST-types

Numbers and percentages of the sequence typed isolates (*n* = 155)			
No of isolates in group n (%)	No of MDR isolates n (%)	ESBL+FQR-MDR isolates n (%)	*p* value
Group A 8(5)	7(88)	2(25)	0.0117
Group B112(8)	10(83)	5(42)	0.0350
Group B2 92(59)	71(77)	33(36)	< 0.0001
Group D 43(28)	36(84)	11(26)	< 0.0001
ST131 71(46)	57(82)	22(31)	< 0.0001
*H*30 35(49)	28(80)	10(29)	< 0.0001
Non *H*30 36(51)	29(81)	12(33)	< 0.0001
ST405 28(18)	24(86)	10(36)	0.0001
ST168 16(10)	14(88)	7(44)	0.0092
ST2913(8)	9(69)	5(38)	0.1156
ST69 5(3)	5(100)	2(40)	0.0384
ST95 2(1)	2(100)	00	
ST31 2(1)	00	00	
ST10 2(1)	1(50)	00	
ST4482(1)	1(50)	00	
ST892(1)	2(100)	1(50)	0.2482
ST7032(1)	2(100)	00	
ST910 1(1)	1(100)	00	
ST5451(1)	00	00	
ST9711(1)	00	00	
ST1531(1)	00	00	
ST1521(1)	1(100)	00	
ST121(1)	1(100)	00	
ST8381(1)	0(100)	00	
NSC3(2)	2(67)	2(67)	> 0.9999

Phylogenetic and sequence type distribution of co-resistance among uropathogenic *E. coli* (*N* = 155). The *p* values were calculated by comparing total number of MDR producers and ESBL producers FQR MDR

### MDR among ST131 strains

pt?>Significant number of the isolates assigned to the phylogenetic group B2 and D were multi-drug resistant (Table 2). Similarly, among different STs including ST131, ST405, ST168, ST29, ST69 and ST89, significant number of the isolates were multi-drug resistant (Table 2). The tendency of ESBL production and the fluoroquinolone resistance was relatively higher among ST131 isolates and majority of these isolates were multi-drug resistant (Table 2). Resistance against nitrofurantoin was significantly higher among ST131 isolates in comparison to the other sequence types, whereas one  of the frequently prevalent sequence types, ST168 strains were significantly resistant to levofloxacin (Table 3). Resistance against carbapenemes has not been evaluated for the scrutinized strains in this study; hence it is beyond the scope of this discussion.

**Table 3:** Chi-squareddistribution of ESBL factors and antibiotic resistance in different ST-types

No (%) of the isolates																						
ST131; n(%)					Other STs; n (%)																	
Resistance traits	All isolates (*n* = 155)	All ST131 (*n* = 71)	Non *H*30 (*n* = 35)	*H*30 (*n* = 36)	ST405 (*n* = 28)	ST168 (*n* = 16)	ST29 (*n* = 13)	ST69 (*n* = 5)	ST95 (*n* = 2)	ST31 (*n* = 2)	ST10 (*n* = 2)	ST448 (*n* = 2)	ST89 (*n* = 2)	ST703 (*n* = 2)	ST910 (*n* = 1)	ST545 (*n* = 1)	ST971 (*n* = 1)	ST153 (*n* = 1)	ST152 (*n* = 1)	ST12 (*n* = 1)	ST838 (*n* = 1)	NSC (*n* = 3)
ESBL phenotypes	65(42)	30(42)	15(43)	15(42)	13(46)	8(50)	7(54)	2(40)	00	00	1(50)	00	1(50)	00	00	00	00	00	1(100)	00	00	2(67)
*bla*CTX-M-15	57(39)	27(38)	12(34)	15(42)	11(39)	8(50)	6(46)	2(40)	00	00	1(50)	00	00	00	00	00	00	00	00	00	00	2(67)
*blaT*EM	23(15)	8(11)	5(14)	3(8)	5(18)	5(31)	3(23)	2(40)	00	00	1(50)	00	1(50)	00	00	00	00	00	1(100)	00	00	00
*bla*SHV	6(4)	3(4)	1(3)	2(6)	1(4)	2(13)	00	00	00	00	00	00	00	00	00	00	00	00	00	00	00	00
*bla*OXA	10(6)	6(8)	3(9)	3(8)	3(11)	1(6)	00	00	00	00	00	00	00	00	00	00	00	00	00	00	00	00
*bla*PSE	1(0.6)	00	00	00	00	00	00	00	00	00	00	00	00	00	1(100)	00	00	00	00	00	00	00
Piperacillin tazobactam	7(5)	3(4)	2(6)	0(0)	3(11)	2(13)	00	00	1(50)	00	00	00	00	00	00	00	00	00	00	00	00	00
Ceftazidime	96(62)	43(61)	20(57)7	23(64)	20(71)	10(63)	9(69)	5(100)	1(50)	00	00	00	1(50)	2(100)	1(100)	00	00	00	00	1(100)	00	00
Cefotaxime	101(65)	46(65)	22(63)	24(67)	20(71)	10(63)	9(69)	5(100)	2(100)	00	1(50)	1(50)	1(50)	2(100)	1(100)	00	00	00	1(100)	00	00	00
Ceftriaxone	99(64)	46(65)	25(71)	21(58)	21(75)	10(63)	9(69)	2(40)	1(50)	00	00	1(50)	1(50)	1(50)	1(100)	1(100)	00	00	1(100)	1(100)	1(100)	2(67)
Ciprofloxacin	95(61)	45(63)	23(66)	22(61)	18(64)	13(81)	6(46)	4(80)	2(100)	1(50)	1(50)	1(50)	00	2(100)	00	00	00	00	00	00	00	2(67)
Levofloxacin	97(63)	45(63)	24(69)	21(58)	19(68)	14^*^(88)	6(46)	4(80)	2(100)	1(50)	2(100)	00	2(100)	00	00	00	00	00	00	00	00	2(67)
Amikacin	7(5)	4(6)	2(6)	2(6)	00	1(6)	1(8)	1(20)	00	00	00	00	00	00	00	00	00	00	00	00	00	00
Gentamicin	47(30)	21(30)	11(31)	10(28)	10(36)	5(31)	3(23)	1(20)	2(100)	00	1(50)	1(50)	00	2(100)	00	00	00	00	00	00	00	1(33)
Amoxicillin-clavulanic acid	111(72)	48(68)	25(71)	23(64)	22(79)	10(63)	9(69)	2(40)	00	00	00	1(50)	1(50)	1(50)	1(100)	1(100)	00	00	1(100)	1(100)	1(100)	2(67)
Trimethoprim sulfonamides	130(84)	61(86)	30(86)	31(86)	24(86)	13(81)	10(77)	4(80)	2(100)	1(50)	1(50)	1(50)	2(100)	2(100)	1(100)	1(100)	1(100)	1(100)	1(100)	1(100)	00	3(100)
Nitrofurantoin	9(6)	9(13)^***^	6(17)^***^	3(8)	00	00	00	00	00	00	00	00	00	00	00	00	00	00	00	00	00	00
Fosfomycin	15(10)	9(13)	3(9)	6(17)	00	2(13)	00	00	00	1(50)	00	1(50)	1(50)	00	00	00	00	00	1(100)	00	00	00

Distribution of antibiotic resistance of uropathogenic *E. coli* (*n* = 155) among different sequence types. The *p* values were calculated by comparing individual STswith each other. The table correlates different traits in vertical columns among different sequence types. The percentages were calculated with reference to total number of sequence types

*P < 0.05, *** *P* ≤ 0.001

### Occurrence of β-lactamases among ST131

Overall, occurrence of ESBL was higher among clonal group ST131, constituting 42% of the total ESBL phenotypes (Table 3). Moreover, 78% of the ESBL phenotypes showed resistance to at least one fluoroquinolone and 95% were resistant to at least one cephalosporin. The occurrence of ESBL genes remained as follows, *bla*_*-*CTX-M-15_, 57(39%), *bla*_-TEM_ 23(15%) and *bla*_-SHV_ 6(4%). Prevalence of other β-lactamases genes such as *bla*_-OXA_ and *bla*_*-*PSE 1_ remained 6 and 0.6% respectively. In comparison to other sequence types, overall prevalence of β-lactamases genes was higher among ST131 strains, 27(38%) of the *bla*_-CTX-M-15_, followed by *bla*_*-*TEM_ 8(11%), *bla*_*-*SHV_ 3(4%) and *bla*_*-OXA*_ *6(8%).* Presence of *bla*_-CTX-M-15_ was highest (100%) among ST131 *H*30*-*O25b and 91% of the *bla*_-CTX-M-15_ positive ST131 *H30-*O25b isolates were resistant to fluoroquinolones (Data not shown). ESBL producing isolates were frequently found resistant to ceftazidime, cefotaxime, ceftriaxone, ciprofloxacin, levofloxacin, amoxicillin-clavulanic acid and trimethoprim sulfonamides (Table 4).

**Table 4:** Distribution of drug resistance and VF genes among ESBL producers and non ESBLUPEC

Numbers and percentages of the isolates andtheir respective traits				
Resistance traits	All isolates (*n* = 155); n(%)	Non ESBL producers (*n* = 90) n(%)	ESBL producers (*n* = 65) n(%)	*p* value
Piperacillin tazobactam	7(5)	4(4)	3(5)	> 0.9999
Ceftazidime	96(62)	36(40)	60(92)	< 0.0001
Cefotaxime	101(65)	40(44)	61(94)	< 0.0001
Ceftriaxone	99(64)	49(54)	50(77)	0.0040
Ciprofloxacin	95(61)	45(50)	50(77)	0.000684
Levofloxacin	97(63)	48(53)	49(75)	0.0051
Amikacin	7(5)	4(4)	3(5)	0.9597
Gentamicin	47(30)	30(33)	17(26)	0.3373
Amoxicillin-clavulanic acid	111(72)	50(56)	61(94)	< 0.0001
Trimethoprim sulfonamides	130(84)	67(74)	63(97)	0.0002
Nitrofurantoin	9(6)	6(7)	3(5)	0.5900
Fosfomycin	15(10)	8(9)	7(11)	0.6960
*fimH*	155 (100)	90 (100)	65 (100)	> 0.9999
*papA*	20 (13)	12 (13)	8 (12)	0.8509
*papC*	75 (48)	42 (47)	33 (51)	0.6140
*papEF*	37 (24)	18 (20)	19 (29)	0.1834
*papGI*	6 (4)	3 (3)	3 (5)	0.6831
*papGII*	70 (45)	33 (37)	37 (57)	0.0124
*papGIII*	6 (4)	4 (4)	2 (3)	0.6632
*sfa/foc*	20 (13)	11 (12)	9 (14)	0.7660
*Afa*	15 (10)	7 (8)	8 (12)	0.3466
*bmaE*	2 (1)	1 (1)	1 (2)	0.8246
*fyuA*	37 (24)	20 (22)	17 (26)	0.5710
*iutA*	86 (55)	45 (50)	41 (63)	0.1060
*feoB*	76 (49)	40 (44)	36 (55)	0.1788
*kpsmtII*	40 (26)	19 (21)	21 (32)	0.1160
*kpsmtIII*	4 (3)	2 (2)	2 (3)	0.4729
*Usp*	22 (14)	10 (9)	12 (18)	0.1957
*hlyA*	18 (12)	7 (8)	11 (17)	0.0795
*cdtB*	11 (7)	6 (7)	5 (8)	0.8062

Distribution of resistance and virulence traits among ESBL and non-ESBL producing uropathogenic *E. coli* (*N* = 155). The *p* values were calculated by comparing different traits among ESBL producer’s and non-ESBL producers

### Distribution of VF genes among different sequence types

A total of 18 different virulence factors were scrutinized among 155 isolates. Percntages of VF genes were as follows: *fim*H,155(100%), *iut*A86 (55%), *feo*B 76 (49%), *pap*C 75 (48%), *papGII*70 (45%), *kpsMTII* 40 (26%), *pap*EF 37 (24%), *fyu*A 37 (24%), *usp* 22 (14%), *pap*A 20 (13%), *sfa/foc*20 (13%), *hly*A 18 (12%), *afa* 15 (10%), *cdt*B 11 (7%), *papGI* 6 (4%), *papGIII* 6 (4%), *kpsMTIII* 4 (3%) and *bma*E2 (1%). Some virulence factors such as *sfa/foc, fyu*A and *feo*B were detected frequently among ST131 isolates whereas VF-genes *pap*EF*, sfa/foc*and *hly*A were frequently associated with *H*30 sub-clone (Table 5). Overall, virulence genes such as sfa*/foc, fyu*A and *feo*B were associated significantly (*p* < 0.05) with ST131 strains, while *pap*EF had significant presence among clonal group ST131-*H30*.

**Table 5:** Chi-squared distribution of virulence factor genes in different ST-types

Number of the isolates with traits n(%)																						
Traits	Total *n* = 155	ST-131 *n* = 71	Non*H*30 *n* = 35	*H*30 *n* = 36	ST-05 *n* = 28	ST-168 *n* = 16	ST-29 *n* = 13	ST-69 *n* = 5	ST-95 *n* = 2	ST-31 *n* = 2	ST-10 *n* = 2	ST-448 *n* = 2	ST-89 *n* = 2	ST-703 *n* = 2	ST-910 *n* = 1	ST-545 *n* = 1	ST-971 *n* = 1	ST-153 *n* = 1	ST-152 *n* = 1	ST-12 *n* = 1	ST-838 *n* = 1	NSC *n* = 3
*fimH*	155 (100)	71 (100)	35 (100)	36 (100)	28 (100)	16 (100)	13 (100)	5 (100)	2 (100)	2 (100)	2 (100)	2 (100)	2 (100)	2 (100)	1 (100)	1 (100)	1 (100)	1 (100)	1 (100)	1 (100)	1 (100)	3 (100)
*papA*	20 (13)	12 (17)	5 (14)	7 (19)	4 (14)	1 (6)	0	0	0	0	0	1 (50)	0	0	0	0	0	1(100)	0	0	0	1 (33)
*papC*	75 (48)	33 (46)	17 (49)	16 (44)	15 (54)	9 (56)	5 (38)	3 (60)	1 (50)	1 (50)	0	0	1 (50)	1 (50)	1 (100)	0	1 (100)	1 (100)	0	0	1 (100)	2 (67)
*papEF*	37 (24)	20 (28)	7 (20)	13^*^ (36)	7 (25)	3 (19)	3 (23)	1 (20)	0	0	0	1 (50)	0	0	0	1 (100)	1 (100)	0	0	0	0	1 (33)
*papGI*	6 (4)	2 (3)	0	2 (6)	1 (4)	1 (6)	0	1 (20)	0	0	0	0	0	0	0	0	0	0	0	0	0	1 (33)
*papGII*	70 (45)	32 (45)	17 (49)	15 (42)	12 (43)	6 (38)	4 (31)	4 (80)	2 (100)	0	2 (100)	1 (50)	2 (100)	1 (50)	0	1 (100)	0	0	0	1 (100)	0	2 (67)
*papGIII*	6 (4)	4 (6)	1 (3)	3 (8)	1 (4)	1 (6)	0	0	0	0	0	0	0	0	0	0	0	0	0	0	0	0
*sfa/foc*	20 (13)	13* (18)	4 (11)	9^*^ (25)	2 (7)	1 (6)	1 (8)	0	1 (50)	0	1 (50)	0	0	0	0	0	0	0	0	0	0	1 (33)
*afa*	15 (10)	7 (10)	4 (11)	3 (8)	6^*^(21)	1 (6)	1 (8)	0	0	0	0	0	0	0	0	0	0	0	0	0	0	0
*bmaE*	2 (1)	1 (1)	1 (3)	0	0	1 (6)	0	0	0	0	0	0	0	0	0	0	0	0	0	0	0	0
*fyuA*	37 (24)	12^*^(17)	7 (20)	5 (14)	10 (36)	6 (38)	2 (15)	2 (40)	0	0	2(100)	0	0	0	0	0	0	0	1 (100)	1 (100)	0	1 (33)
*iutA*	86 (55)	41 (58)	22^***^ (63)	19 (53)	14 (50)	10 (63)	5 (38)	3 (60)	2 (100)	1 (50)	2 (100)	1 (50)	1 (50)	1 (50)	1 (100)	0	1 (100)	0	1 (100)	0	0	2 (67)
*feoB*	76 (49)	28^*^ (39)	13 (37)	15 (42)	12 (43)	10 (63)	6 (46)	4 (80)	2 (100)	1 (50)	2 (100)	1 (50)	2 (100)	2 (100)	1 (100)	0	1 (100)	0	1 (100)	1 (100)	0	2 (67)
*kpsmtII*	40 (26)	20 (28)	9 (26)	11 (31)	8 (29)	2 (13)	2 (15)	2 (40)	0	0	0	1 (50)	0	0	0	0	0	0	1 (100)	1 (100)	1 (100)	2 (67)
*kpsmtIII*	4 (3)	2 (3)	0	2 (6)	0	0	0	1 (20)	0	0	0	0	0	0	0	1 (100)	0	0	0	0	0	0
*usp*	22 (14)	13 (18)	5 (14)	8 (22)	2 (7)	3 (19)	1 (8)	0	0	0	1 (50)	0	0	0	0	1(100)	0	0	0	0	0	1 (33)
*hlyA*	18 (12)	8 (11)	1 (3)	7^*^ (32)	5 (18)	1 (6)	1 (8)	0	1 (50)	0	0	0	0	0	0	0	0	0	0	1 (100)	0	1 (33)
*cdtB*	11 (7)	4 (6)	1 (3)	3 (8)	4 (14)	1 (6)	0	0	1(50)	0	0	0	0	0	0	0	0	0	0	0	0	1 (33)

Distribution of virulence traits of uropathogenic *E. coli* (*n* = 155) among different sequence types. The *p* values were calculated by comparing individual STs with each other. The table correlates different traits in vertical columns among different sequence types. The percentages were calculated with reference to total number of sequence types

*P < 0.05, ** *P* ≤ 0.01, *** *P* ≤ 0.001

## Discussion

*E. coli* ST131 was reported from three different continents [8]. However, recently it has become the most predominant lineage associated with variety of infections around the globe. ST131 strains have a tendency to harbor ESBL enzymes *bla*-_CTX-M-15_, which play a significant role in mounting resistance against  β-lactam class of antibiotics [8]. Moreover, ST131 strains show remarkable resistance to the fluoroquinolones and demonstrate greater abilities to adhere bladder, kidneys and epithelial cells [4, 5]. In this study, clonal group ST131 was the most prevalent lineage, comprising 46% of the isolates and majority of these isolates (59%) belonged to the phylogenetic group B2. Prevalence of two other lineages ST405 and ST168 were 18 and 10% respectively. Involvement of these lineages in UTIs has been described earlier and recently ST405 was confirmed as an emerging uropathogenic *E. coli* clone in Saudi Arabia [21, 22]. Urinary tract infections caused by *E. coli* pose considerable challenge and are associated with higher morbidity and mortality [5]. Due to their resistance against variety of antibiotics, including β-lactams, aminoglycosides and fluoroquinolones, infections caused by pandemic clonal group ST131 are particularly challenging to treat [23, 24]. In this context, epidemiological significance of a sub group ST131 *H30*-Rx has been well described [5, 25]. In this study, 50% of the ST131 strains carried *H*30 variant of *fim*H gene and 61% belonged to the serogroup O25b. All the isolates belonging to the sub-group ST131-*H30-*O25b carried ESBL *bla*-_CTX-M-15_ however, overall prevalence of these particular strains constituted only 10% of the total isolates. Resistance against fluoroquinolones in ST131 strains remained 60%, that was remarkably higher. For the treatment of UTIs commonly prescribed antibiotics include sulphamethoxazole-trimethoprim and fluoroquinolones. However, due to the emerging resistance to these antibiotics alternative therapeutic choices such as nitrofurantoin, fosfomycin and β-lactam inhibitors can be prescribed.

In this study, prevalence of ESBL genes was higher among ST131 and 90% of these strains were resistant to ceftazidime and cefotaxime. Likewise, resistance to ceftriaxone was confirmed in 77% of these strains. Because of their favorable safety, cephalosporins are considered important therapeutic choice for the treatment of uncomplicated UTIs among pregnant women [26].

Nitrofurantoin is a fluoroquinolone-sparing alternative antibiotic used for the treatment of uncomplicated cystitis [27]. In recent years use of nitrofurantoin has increased steadily, particularly due to the resistance against trimethoprim/sulfamethoxazole and aminopencillins. Contraindication of ciprofloxacin in pregnancy and adverse impact on the gut flora favored the use of nitrofurantoin as an alternative treatment option for UTIs. In this study 13% of the ST-131 strains were resistant to nitrofurantoin.

We found that majority of the isolates belonging to the lineages ST405, ST168, ST29, ST69 and ST89 were multi-drug resistant. Percentage of MDR isolates was particularly higher among fluoroquinolones-resistant ST131 strains. Overall 59% of the isolates belonged to the phylogenetic group B2. A previous study from Pakistan confirmed that 50% of the UPEC isolates belonged to the phylogenetic group B2 [28]. Likewise, another study conducted in Pakistan reported that only 12% of the *E. coli* strains belonged to this phylogenetic group. These findings suggest that prevalence of phylogenetic group B2 may vary across different regions [29]. Few studies conducted previously in this region included phylogentic analysis of UPEC strains.

Phylogenetic group B2 strains are equipped with various VF genes relating to the extra-intestinal infections. These genes include P-fimbriae, S-fimbriae, haemolysin, aerobactin, K1 and K5 antigens and capsular antigen genes [30, 31]. A previous report focusing on the UPEC, in Pakistan described prevalence of various VF genes, including *hlyA, sfaDE, papC,cnf1, eaeA* and *afaBC* [29] While another study conducted on the rectal floral isolates of Pakistani children confirmed that virulence factors such as S-fimbriae, haemolysin, K-1 antigens and class III PapG adhesins were either very rare or completely absent [29]. In current study, UPEC strains of phylogenetic group B2, carried range of virulence factors, including genes for adhesins (*fimH* 100%, *papA* 13%, *papC* 47%, *papEF* 21% *papGI* 3%, *papGII* 40%, * papGIII* 4%*, sfa/foc *14%*, afa* 11%*, bmaE* 1%), toxins (*hlyA* 7%, *cdtB* 7%) iron acquisition system (*iutA* 57%, *feoB*43%*, fyuA* 23%) capsular proteins (*kpsMTII* 26%, *kpsMTIII* 3%) and uropathogenic specific protein (*usp* 14%). We observed that the gene *papGII* was significantly associated with phylogenetic group B2 strains and association of *papGII* with pyelonephritis and bacteraemia in human has been confirmed earlier [32–34]. In the current study, fimbriae associated gene *fimH* was detected among 100% of the UPEC isolates Role of *fimH* in adhesion, invasion and formation of the intracellular bacterial communities (IBCs) has been described previously and its importance in the host pathogen interaction was confirmed by higher vulnerabilities of premenopausal women to UPEC infections [35]. In this study genes related to the adhesins *(papEF, sfa/foc)* and toxins (*hlyA)* were found to be strongly associated with ST131 *H30* sub-clone. Recently *hly*A in interaction with natural killer (NK) cells of urinary bladder was described [36]. Likewise, we witnessed significant association of the iron acquisition genes (*(fyuA *and *feoB)* with ST131 lineage. The importance of gentic factors related to the iron acquisition system was shown by strong upregulation of these genes during UTIs [37]. Generally, *E. coli* strains causing UTI share similar properties in terms of phylogeny, sero-grouping and VF genes. However, other than genetic attributes of the virulence strains, host factors may play important role in the outcome of infection [38].

## Conclusion

In conclusion it is the first report that highlights MDR ST131 as a predominant linage associated with UTI in Pakistan. ST131 and other scrutinized sequence types having MDR status among UTI isolates in Pakistan indicate considerable constraints on the empirical choice for the treatment of UTI. Alternative therapies and identification of effective prevention strategies–including antibiotic stewardship – are needed. As antibiotic resistance can be transferred from UPEC to other pathogens, more judicious use of antibiotics is required.

## Data Availability

The datasets used and/or analyzed during the current study available from the corresponding author on reasonable request.

## Abbreviations

ESBL: Extended spectrum beta lactamases
IBCs: Intracellular bacterial communities
MDR: Multidrug resistance
NK: Natural killer
ST: Sequence type
UPEC: Uropathogenic *E. coli*
UTI: Urinary tract infections
VF: Virulence factors

## References

[1] Dehbanipour R, Rastaghi S, Sedighi M, Maleki N, Faghri J (2016). High prevalence of multidrug-resistance uropathogenic *Escherichia coli* strains, Isfahan, Iran. J Nat Sci Biol Med.

[2] Mittal S, Sharma M, Chaudhary U (2015). Biofilm and multidrug resistance in uropathogenic Escherichia coli. Pathog Glob Health.

[3] Tanvir R, Hafeez R, Hasnain S (2012). Prevalence of multiple drug resistant *Escherichia coli* in patients of urinary tract infection registering at a diagnostic laboratory in Lahore Pakistan. Pak J Zool.

[4] Johnson JR, Tchesnokova V, Johnston B, Clabots C, Roberts PL, Billig M, Riddell K, Rogers P, Qin X, Butler-Wu S (2013). Abrupt emergence of a single dominant multidrug-resistant strain of Escherichia coli. J Infect Dis.

[5] Petty NK, Zakour NLB, Stanton-Cook M, Skippington E, Totsika M, Forde BM, Phan M-D, Moriel DG, Peters KM, Davies M (2014). Global dissemination of a multidrug resistant *Escherichia coli* clone. Proc Natl Acad Sci U S A.

[6] Shahzad N, Aslam B, Hussain I, Ijaz M, Rasool MH, Tasneem F, Hamid T, Tayyeb A, Hussain T. Distribution and Phylogenetic Analysis of Bacterial Isolates from Urinary Tract Infection Patients of Pakistan. Pak J Zool. 2016;48(6):1925–30.

[7] Johnson JR, Johnston B, Clabots C, Kuskowski MA, Castanheira M (2010). *Escherichia coli* sequence type ST131 as the major cause of serious multidrug-resistant E. coli infections in the United States. Clin Infect Dis.

[8] Nicolas-Chanoine M-H, Bertrand X, Madec J-Y (2014). *Escherichia coli* ST131, an intriguing clonal group. Clin Microbiol Rev.

[9] Johnson JR, Johnston B, Thuras P, Launer B, Sokurenko EV, Miller LG (2016). *Escherichia coli* Sequence Type 131 H30 Is the Main Driver of Emerging Extended-Spectrum-β-Lactamase-Producing *E. coli* at a Tertiary Care Center. mSphere.

[10] Johnson JR, Menard M, Johnston B, Kuskowski MA, Nichol K, Zhanel GG (2009). Epidemic clonal groups of *Escherichia coli* as a cause of antimicrobial-resistant urinary tract infections in Canada, 2002 to 2004. Antimicrob Agents Chemother.

[11] Cagnacci S, Gualco L, Debbia E, Schito GC, Marchese A (2008). European emergence of ciprofloxacin-resistant *Escherichia coli* clonal groups O25: H4-ST 131 and O15: K52: H1 causing community-acquired uncomplicated cystitis. J Clin Microbiol.

[12] Hefzy EM, Hassuna NA (2017). Fluoroquinolone-resistant sequence type 131 subgroups O25b and O16 among Extraintestinal *Escherichia coli* isolates from community-acquired urinary tract infections. Microb Drug Resist.

[13] Clinical and Laboratory Standards Institute (2014). Performance standards for antimicrobial susceptibility testing: 24th informational supplement. . Document M100–S24.

[14] Clermont O, Bonacorsi S, Bingen E (2000). Rapid and simple determination of the*Escherichia coli* phylogenetic group. Appl Environ Microbiol.

[15] Weissman SJ, Johnson JR, Tchesnokova V, Billig M, Dykhuizen D, Riddell K, Rogers P, Qin X, Butler-Wu S, Cookson BT (2012). High-resolution two-locus clonal typing of extraintestinal pathogenic Escherichia coli. Appl Environ Microbiol.

[16] Tchesnokova V, Billig M, Chattopadhyay S, Linardopoulou E, Aprikian P, Roberts PL, Skrivankova V, Johnston B, Gileva A, Igusheva I (2013). Predictive diagnostics for *Escherichia coli* infections based on the clonal association of antimicrobial resistance and clinical outcome. J Clin Microbiol.

[17] Clermont O, Dhanji H, Upton M, Gibreel T, Fox A, Boyd D, Mulvey MR, Nordmann P, Ruppé E, Sarthou JL (2009). Rapid detection of the O25b-ST131 clone of *Escherichia coli* encompassing the CTX-M-15-producing strains. J Antimicrob Chemother.

[18] del Castillo BR, Vinué L, Román EJ, Guerra B, Carattoli A, Torres C, Martínez-Martínez L (2013). Molecular characterization of multiresistant *Escherichia coli* producing or not extended-spectrum β-lactamases. BMC Microbiol.

[19] Bush KTP JG (2015). CTX-M-type Beta-lactamases. Lahey Clinic.

[20] Yun KW, Kim HY, Park HK, Kim W, Lim IS (2014). Virulence factors of uropathogenic *Escherichia coli* of urinary tract infections and asymptomatic bacteriuria in children. J Microbiol Immunol Infect.

[21] Alghoribi MF, Gibreel TM, Farnham G, Al Johani SM, Balkhy HH, Upton M (2015). Antibiotic-resistant ST38, ST131 and ST405 strains are the leading uropathogenic *Escherichia coli* clones in Riyadh, Saudi Arabia. J Antimicrob Chemother.

[22] Peirano G, Pitout JD (2010). Molecular epidemiology of *Escherichia coli* producing CTX-M β-lactamases: the worldwide emergence of clone ST131 O25: H4. Int J Antimicrob Agents.

[23] Banerjee R, Robicsek A, Kuskowski MA, Porter S, Johnston BD, Sokurenko E, Tchesnokova V, Price LB, Johnson JR (2013). Molecular epidemiology of *Escherichia coli* sequence type 131 and its H30 and H30-Rx subclones among extended-spectrum-β-lactamase-positive and-negative E. coli clinical isolates from the Chicago region, 2007 to 2010. Antimicrob Agents Chemother.

[24] Lau SH, Reddy S, Cheesbrough J, Bolton FJ, Willshaw G, Cheasty T, Fox AJ, Upton M (2008). Major uropathogenic *Escherichia coli* strain isolated in the northwest of England identified by multilocus sequence typing. J Clin Microbiol.

[25] Johnson JR, Clermont O, Johnston B, Clabots C, Tchesnokova V, Sokurenko E, Junka AF, Maczynska B, Denamur E (2014). Rapid and specific detection, molecular epidemiology, and experimental virulence of the O16 subgroup within *Escherichia coli* sequence type 131. J Clin Microbiol.

[26] Nicolle L (1996). Management of asymptomatic UTIs in women. Medscape Womens Health.

[27] Hooton TM (2003). Fluoroquinolones and resistance in the treatment of uncomplicated urinary tract infection. Int J Antimicrob Agents.

[28] Bashir S, Haque A, Sarwar Y, Ali A, Anwar MI (2012). Virulence profile of different phylogenetic groups of locally isolated community acquired uropathogenic *E. coli* from Faisalabad region of Pakistan. Ann Clin Microbiol Antimicrob.

[29] Nowrouzian F, Östblom A, Wold A, Adlerberth I (2009). Phylogenetic group B2 *Escherichia coli* strains from the bowel microbiota of Pakistani infants carry few virulence genes and lack the capacity for long-term persistence. Clin Microbiol Infect.

[30] Abdi HA, Rashki A (2014). Comparison of virulence factors distribution in uropathogenic E. coli isolates from phylogenetic groups B2 and D. Int J Enteric Pathog.

[31] Er DK, Dundar D, Uzuner H, Osmani A (2015). Relationship between phylogenetic groups, antibiotic resistance and patient characteristics in terms of adhesin genes in cystitis and pyelonephritis isolates of Escherichia coli. Microb Pathog.

[32] Johnson JR (1998). *papG* alleles among *Escherichia coli* strains causing urosepsis: associations with other bacterial characteristics and host compromise. Infect Immun.

[33] Johnson JR, Kuskowski MA, Gajewski A, Soto S, Horcajada JP, de Anta MTJ, Vila J (2005). Extended virulence genotypes and phylogenetic background of *Escherichia coli* isolates from patients with cystitis, pyelonephritis, or prostatitis. The J Infect Dis..

[34] Otto G, Sandberg T, Marklund B-I, Ulleryd P, Svanborg C (1993). Virulence factors and pap genotype in *Escherichia coli* isolates from women with acute pyelonephritis, with or without bacteremia. Clin Infect Dis.

[35] Foxman B, Brown P (2003). Epidemiology of urinary tract infections: transmission and risk factors, incidence, and costs. Infect Dis Clin N Am.

[36] Gur C, Coppenhagen-Glazer S, Rosenberg S, Yamin R, Enk J, Glasner A, Bar-On Y, Fleissig O, Naor R, Abed J (2013). Natural killer cell-mediated host defense against uropathogenic E. coli is counteracted by bacterial hemolysinA-dependent killing of NK cells. Cell Host Microbe.

[37] Hagan EC, Lloyd AL, Rasko DA, Faerber GJ, Mobley HL (2010). *Escherichia coli* global gene expression in urine from women with urinary tract infection. PLoS Pathog.

[38] Takahashi A, Kanamaru S, Kurazono H, Kunishima Y, Tsukamoto T, Ogawa O, Yamamoto S (2006). *Escherichia coli* isolates associated with uncomplicated and complicated cystitis and asymptomatic bacteriuria possess similar phylogenies, virulence genes, and O-serogroup profiles. J Clin Microbiol.

